# A novel moving phantom insert for image quality assessment in magnetic resonance imaging

**DOI:** 10.1016/j.phro.2025.100742

**Published:** 2025-03-07

**Authors:** Frédérique P.D. van Gameren, Pim T.S. Borman, Cornelis A.T. van den Berg, Mike Cole, Grant R. Koenig, Martin F. Fast, Astrid L.H.M.W. van Lier

**Affiliations:** a University Medical Center Utrecht, Department of Radiotherapy, Utrecht, the Netherlands; b IBA Quasar, Modus Medical Devices, London, Ontario, Canada

**Keywords:** MRIgRT, Motion compensation, Quality assurance, Phantom design, MRI

## Abstract

Dedicated motion compensated Magnetic Resonance Imaging (MRI) for radiotherapy treatment planning promises to mitigate motion effects on imaging. We demonstrate a novel insert for an MRI safe motion phantom, which enables quality assurance of these image strategies. The capability to analyse apparent slice thickness, positional accuracy and motion blur is demonstrated for scenarios with and without motion. A respiratory-compensated scan with a 4 mm trigger-window and 16 mm peak-to-peak (p2p) motion showed a +5.0% deviation from the nominal 2 mm slice thickness. In contrast, a non-compensated scan with 4 mm p2p motion showed a +77.5% deviation, illustrating the effectiveness of motion compensation.

## 1 Introduction

Respiratory motion is a prominent source of uncertainty in both Magnetic Resonance Imaging (MRI) and external beam radiotherapy delivery for thoracic and abdominal lesions [1]. In one respiratory cycle, a lung tumor moves typically around 20 mm peak-to-peak (p2p) but can move beyond 30 mm p2p [2]. Motion compensated imaging techniques can be used to suppress motion artefacts and acquire sharp images that can be used for treatment planning [[3], [4], [5], [6]]. For radiotherapy purposes it is critical that these motion compensated imaging techniques lead to geometrically correct representations of the anatomy [7]. This means that the position and extent of the tumor and surrounding organs at risk are accurately shown despite motion. Appropriate quality assurance (QA) is warranted if radiotherapy dedicated MRI sequences are used [8]. However, currently available phantoms, e.g. the MRI-American College of Radiology (ACR) phantom, are not capable of performing this task as they operate under static conditions [9,10].

In this work we present a phantom insert which we designed to quantify apparent slice thickness, positional accuracy and motion blur [11] of MRI scans acquired while moving the phantom. Apparent slice thickness and motion blur measure how well a volume can be visualized in case of in-plane or though-plane motion, while positional accuracy measures the position of the measured volume with respect to the respiratory phases.

## 2 Materials and methods

### 2.1 Image acquisitions

MRI scans without motion compensation, with navigator triggering and retrospective motion correction were performed to highlight the performance of the phantom (see Supplementary Table S1 for details).

#### 2.1.1 MR-simulation scans

We tested the phantom insert on a 1.5 T MR-sim (Ingenia, Philips Healthcare, the Netherlands) using a standard T1-weighted Cartesian 3D Ultrafast Gradient Echo (T1w GRE) sequence (*sequence 1*) and an investigational motion compensated T2-weighted PROPELLER Multi-Slice Turbo Spin Echo sequence (T2w PROPELLER) with 1D respiratory navigator (1D-RNAV) triggering in end-exhale [[5], [12]] (*sequence 2*).

*Apparent slice thickness* measurements were made using nominal slice thicknesses of 2.0–5.0 mm without moving the insert. Sequence 1 was repeated for a nominal slice thickness of 2.0 mm with increasing p2p motion (cosine^4^ wave; 2, 4, 6, 8 mm p2p amplitude; 0.25 Hz). Sequence 2 was repeated for nominal slice thicknesses of 2.0–5.0 mm with motion (cosine^4^ wave, 16 mm p2p amplitude, 0.22 and 0.25 Hz) and varying trigger-windows (TWs) (4, 6 and 8 mm).

For *positional accuracy* measurements, we first scanned without moving the insert and repeated these scans with different p2p motion amplitudes (cosine^4^ wave, 2–20 mm p2p amplitude, 0.25 Hz). We also tested the phantom for sequence 2 with motion (cosine^4^, 16 mm p2p amplitude, 0.22 and 0.25 Hz) and ranging TWs (4, 6 and 8 mm).

For *motion blur* measurements, we performed the same scans as for positional accuracy. Additionally, we performed the untriggered scans (*sequence 2*) for 2, 6, 10, 14 and 18 mm p2p amplitudes each three times.

#### 2.1.2 MR-linac scans

We tested the phantom insert on a 1.5 T Unity MR-linac (Elekta AB, Sweden) using an investigational motion compensated sequence (*sequence 2*) and a selection of clinically used sequences (*sequences 3*–*5*): a T2-weighted Cartesian 3D velocity-based 1D-RNAV triggered sequence (*sequence 3*) [13], a T1-weighted Stack-of-stars 3D Vane XD sequence [[14], [15]] (*sequence 4*) and a standard (non-motion compensated) T2-weighted Cartesian 3D sequence (*sequence 5*).

For all scans, we used a motion trajectory of a cosine^4^ with a 16 mm p2p amplitude and a 0.25 Hz frequency, which represents a realistic respiratory motion trace [2].

### 2.2 Phantom design and analysis

The following requirements were specified for the phantom insert: (1) The insert must be compatible with the QUASAR MRI4D Motion Phantom (IBA Quasar, Canada) which allows for motion of the insert during MRI acquisitions. (2) The insert must hold QA components to analyze *apparent slice thickness, positional accuracy* and *motion blur*. (3) The insert must have a volume to obtain the 1D-RNAV signal with enough distance between the 1D-RNAV and QA components to prevent interference with the imaging plane. (4) Orientation of the QA components must allow analysis over all three cardinal orientations.

**Fig. 1:**
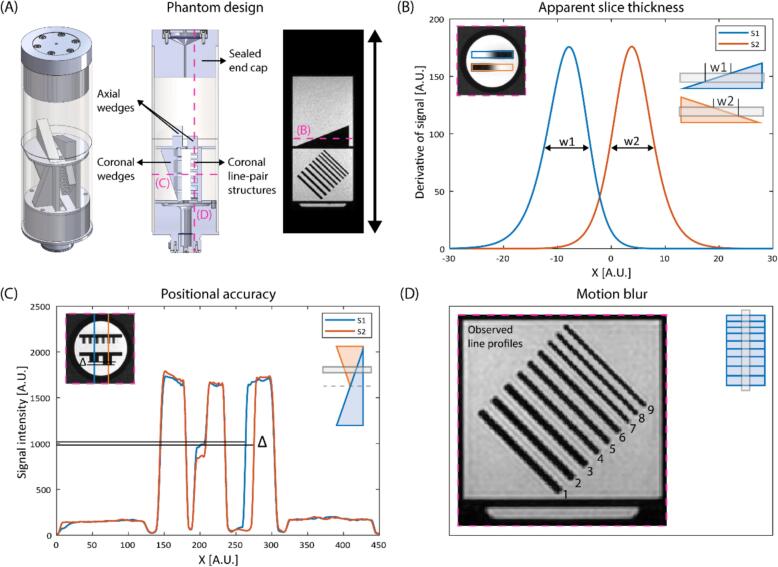
(A) 3D view, sagittal cut-away view and a coronal MRI of the phantom showing the sealed end cap, wedges and line-pair structures. (B) Method to determine the apparent slice thickness by using the axial wedges, (C) to determine the positional accuracy by using the coronal wedges and (D) to determine the motion blur by using the coronal line-pair structures.

The dedicated phantom insert consists of a mineral-oil-filled compartment into which several components such as wedges and line-pair structures are submerged (see Supplementary Table S2 for dimensions). The fluid is chosen such that the T2 is comparable to values of tissue in the human body [16] and with a short T1 to prevent flow artefacts (T1 = 168.7 ms, T2 = 57.5 ms at 1.5 T). The phantom is closed by a flat sealed end cap to allow 1D-RNAV placement (Fig. 1A).

### 2.3 Apparent slice thickness

For *apparent slice thickness* measurements, the axial wedge-shaped QA components were used. In the axial images, a profile along the wedge gray scale gradient was manually drawn. The slice thickness is calculated by deriving the Full Width Half Maximum (FWHM) of the derivative of this signal profile. The use of two wedges resulted in two FWHM values ($w_{1}$, $w_{2}$), from which the apparent slice thickness (ST) was calculated: ST=12·w1+w2 $\cdot \tan\alpha_{\text{wedge}}$[11] (Fig. 1B). This QA component design is similar to the ACR phantom. However, the quantification is slightly different [9].

### 2.4 Positional accuracy

For *positional accuracy* measurements, the coronal wedge-shaped QA components were used. The relative slice position is obtained by measuring the length difference of the right and left vertical black bars. The deviation in length is determined by analyzing the difference in length (Δ) of the black bars (i.e. wedge cross sections) as seen in the axial images (Fig. 1C). Initially, the scan is performed without motion with the insert at a 0 mm offset position ($\Delta_{\mathrm{static}}$). The measurements are repeated with the insert in motion ($\Delta_{\mathrm{moving}}$). The relative slice position (*dz*) is derived by

$\mathrm{dz}=\left(\Delta_{\mathrm{static}}-\Delta_{\mathrm{moving}}\right)/\left(2\cdot \tan\left(\alpha_{\mathrm{wedge}}\right)\right)$. The observed deviation with respect to the average position over time (ie. integrated cosine^4^) of the phantom is given. For the triggered scans, the predicted average position within the TW was calculated and compared to the observed positions. This QA component design and method is comparable to the ACR phantom. However, in that case the respective image is compared to the planned image [9].

### 2.5 Motion blur

For *motion blur* assessment, the coronal line-pair QA components were used. In the coronal images, the parallel line profiles were observed by two independent observers. The number of line profiles that were visible were counted (Fig. 1D).

## 3 Results

### 3.1 MR-simulation scans

#### 3.1.1 Apparent slice thickness

**Fig. 2:**
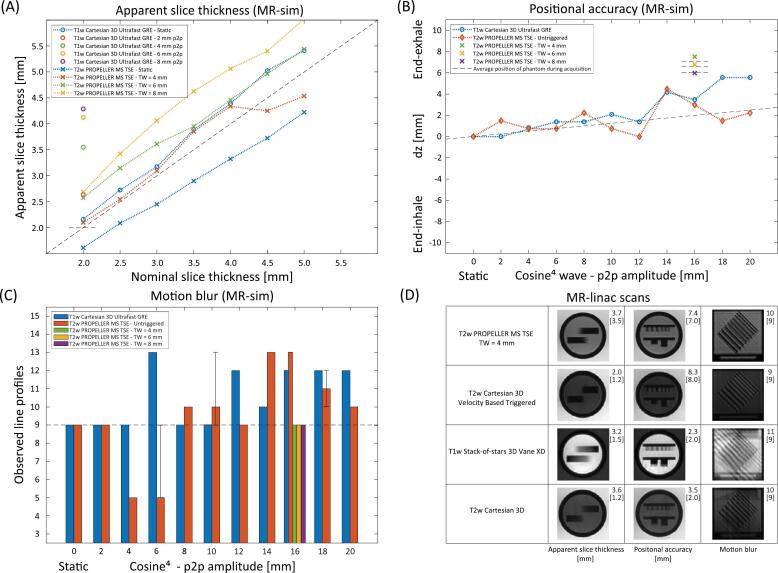
Results for (A) apparent slice thickness results, (B) positional accuracy and (C) motion blur acquired on a 1.5 T MR-sim. (B) The average position of the phantom is depicted with a dashed line. The average position without triggering is simply the average position of the phantom over time, which depends on the p2p amplitude of the cosine^4^ wave. For the triggered T2w PROPELLER scans, the anticipated average positions of the phantom during acquisition within the trigger-window were +7.1, +6.6 and +6.0 mm for TW’s of 4, 6 and 8 mm, respectively. (D) Results for apparent slice thickness, positional accuracy and motion blur of scans acquired on a 1.5 T MR-linac.

For T1w GRE scans without motion, we observed a slight overestimation of the apparent slice thickness with deviations ranging from +5.8% to +11.7% from the nominal slice thickness. For static T2w PROPELLER scans, we observed an underestimation of apparent slice thickness with deviations ranging from –15.5% to –19.4%. For T2w PROPELLER scans with motion and a TW of 4 mm, we observed an increased ST, with deviations ranging from –9.0% to +10.4%. For T2w PROPELLER scans with a TW of 6 mm, the deviations further increased from +8.8% to +28.8% and for a TW of 8 mm the largest deviations were found (+20.0% to +37.0%). The results of T1w GRE scans with motion showed the same trend: in that case larger p2p motion resulted in larger deviations between the apparent and nominal slice thickness (Fig. 2A).

#### 3.1.2 Positional accuracy

For T1w GRE scans, the positional accuracy deviated between –0.3 mm to +3.3 mm from the average position of the corresponding cosine^4^ motion trajectory. Scans performed while moving the phantom with larger p2p motion amplitudes resulted in larger deviations from the average position respectively. The untriggered T2w PROPELLER scans deviated between –1.5 mm to +2.7 mm from the average position of the phantom insert. Triggered T2w PROPELLER scans with increasing TWs (4, 6 and 8 mm) showed an increasing positioning deviation for smaller TWs (respectively, +7.4, +6.7 and +6.0 mm) towards the end-exhale position. In that case, the triggered T2w PROPELLER scans deviated +0.4, +0.1 and –0.1 mm from the average position of the phantom predicted from the set TW (Fig. 2B).

#### 3.1.3 Motion blur

The T1w GRE motion scans showed increasing motion blur relative to the static scan for increasing p2p motion amplitudes. The amount of observed line profiles ranged from 9 to 13. From p2p amplitudes of 6 mm (13 line profiles observed) and higher, it became more difficult to distinguish the separate line-pair structures. For the untriggered T2w PROPELLER scans, the same trend occurred. The scans with p2p amplitudes of 2, 6, 10, 14 and 18 mm were each performed three times and resulted in images with different effects of the motion blur, hence the range of observed line profiles for scans with 6 mm p2p (5–9), 10 mm p2p (9–13) and 18 mm p2p (10–12). For the triggered T2w PROPELLER scans with increasing TW widths (4, 6 and 8 mm), the independent observers counted 9 line profiles each. The image quality was less affected by motion blur compared to the scans with the same motion amplitude (16 mm p2p) without triggering or motion compensation (Fig. 2C).

### 3.2 MR-linac scans

Fig. 2D shows the results of the MR-linac scans. The *apparent slice thickness* deviated +6.4%, +68.8%, +114.2% and +196.9% (+0.2, +0.8, +1.7 and +2.4 mm) from the nominal slice thickness for sequences 2, 3, 4 and 5. The *relative slice positions* were +7.4, +8.3, +2.3 and +3.5 mm for sequences 2, 3, 4 and 5, respectively. All shifted towards the end-exhale position, which was positioned at +8.0 mm. The triggered T2w PROPELLER scan deviated +0.4 mm from the average position of the phantom within the set TW (+7.1 mm). The *motion blur* strongly depended on the sequence. The amount of line profiles counted were 10, 11, 9 and 10 for sequences 2, 3, 4 and 5.

## 4 Discussion

In this study we showed a novel prototype phantom insert that allows for analysis of *apparent slice thickness, positional accuracy* and *motion blur* of MRI scans performed during motion. The presented phantom and analysis methods can be used to guide protocol optimization and quality assurance of MRI scans for radiotherapy treatment purposes performed under respiratory motion, e.g. for abdominal and thoracic lesions. The insert has some limitations. Firstly, it does not contain anthropomorphic structures and its intended use does not include comprehensive testing of the full MRI-guided radiotherapy workflow. Secondly, the T1 value of the mineral oil in the insert (168.7 ms) is considerably shorter than in vivo values [16]. This prevents flow artefacts within the phantom, but could also result in unintentional signal nulling in fat suppressed scans. Lastly, no accepted tolerances are specified for the observed metrics, as the tolerance levels will depend on the treatment strategy (e.g. gating, tracking) and PTV-margins.

For the non-triggered scans, performed on the MR-sim, increasingly larger deviations from the *apparent slice thickness* were observed for larger p2p motion amplitudes, indicating that information from a width larger than the nominal slice thickness is depicted in this image. For *positional accuracy* tests, it was demonstrated that larger deviations from the average position of the phantom for motion trajectories with larger p2p motion amplitudes for non-triggered scans are observed. Furthermore, we observed for triggered T2w PROPELLER scans an increasing positioning deviation towards end-exhale for reducing TWs, corresponding to the average position of the phantom within the set TW. For *motion blur* scans, it became more difficult to distinguish the separate line profiles for increasing p2p motion amplitudes, highlighting the poorer image quality of those scans. Furthermore, triggered T2w PROPELLER scans showed less *motion blur* compared to untriggered T2w PROPELLER or non-motion compensated sequences moving with the same motion trajectory. This behavior is expected, as for small TWs, data acquisition should only be performed closer to the end-exhale position, where less motion is present.

The *motion blur* scans moving with p2p amplitudes of 2, 6, 10, 14 and 18 mm were each performed three times and resulted in different images. This can be explained by the semi-random interplay effect between the sampling of the separate blades in a PROPELLER acquisition and the timing of the motion pattern which results in different effects on motion blur. For the triggered scans, this semi-random interplay is no longer present. However, in this situation we observed constructive interference between certain motion patterns and a particular TW (6 mm), leading to an unexpectedly larger apparent slice thickness (see Supplementary Fig. S1). In retrospect, this interference could be explained by the fact that the time the phantom resided in the TW coincided exactly with the time needed to acquire one blade for all slices. Therefore, each slice was always acquired at the same phase of the motion pattern which, in this case, was the phase of the cosine^4^ in which most motion was present. Slightly changing the motion frequency to prevent this interference directly impacted the observed apparent slice thickness, which was more in line with the expected value. This finding showcases that the phantom can also be a valuable tool to investigate the impact of motion frequencies on image quality, which might find its application in optimization of predefined breathing patterns for patients [17]. These interplay effects also show that for proper clinical quality assurance repeated imaging is warranted, as well as varying phantom motion patterns. In this article we only performed repeated measurements on selected scans to show this behavior.

As is highlighted in the MR-linac scan set, the experimental triggered T2w PROPELLER scans with TWs of 4, 6 and 8 mm performed good compared to the clinical sequences for *apparent slice thickness* and *image blur.* This shows that this sequence provides information with fewer artifacts and more accurately reflects the actual tumor dimensions compared to the other sequences. Furthermore, the *positional accuracy* tests showed that a position near end-exhale is depicted for triggered T2w PROPELLER scans, which aligns with an exhale gated radiotherapy workflow [18]. We suggest that prior to clinical introduction more motion patterns are tested to further investigate robustness of this method.

## CRediT authorship contribution statement

**Frédérique P.D. van Gameren:** Conceptualization, Methodology, Software, Validation, Formal analysis, Investigation, Data curation, Visualization, Writing – original draft. **Pim T.S. Borman:** Conceptualization, Software. **Cornelis A.T. van den Berg:** Conceptualization, Funding acquisition, Project administration, Supervision. **Mike Cole:** Conceptualization, Methodology, Resources, Writing – review & editing. **Grant R. Koenig:** Conceptualization, Methodology, Resources. **Martin F. Fast:** Conceptualization, Methodology, Validation, Formal analysis, Project administration, Supervision, Visualization, Writing – review & editing. **Astrid L.H.M.W. van Lier:** Conceptualization, Methodology, Software, Validation, Formal analysis, Data curation, Project administration, Supervision, Visualization, Writing – review & editing.

## Declaration of competing interest

The authors declare that they have no known competing financial interests or personal relationships that could have appeared to influence the work reported in this paper.

## References

[1] Korreman S., Persson G., Nygaard D., Brink C., Juhler-Nøttrup T. (2012). Respiration-correlated image guidance is the most important radiotherapy motion management strategy for most lung cancer patients. Int J Radiat Oncol Biol Phys.

[2] Langen K., Jones D. (2001). Organ motion and its management. Int J Radiat Oncol Biol Phys.

[3] Zaitsev M., Maclaren J., Herbst M. (2015). Motion artifacts in MRI: A complex problem with many partial solutions. J Magn Reson Imaging.

[4] van Heeswijk R.B., Bonanno G., Coppo S., Coristine A., Kober T., Stuber M. (2012). Motion compensation strategies in magnetic resonance imaging. Crit Rev Biomed Eng.

[5] Pipe J.G. (1999). Motion correction with PROPELLER MRI: application to head motion and free-breathing cardiac imaging. Magn Reson Med.

[6] Kapur S., Jana M., Gupta L., Bhalla A.S., Naranje P., Gupta A.K. (2021). Chest MRI using Multivane-XD, a novel T2-Weighted free breathing MR sequence. Current Problems Diagn Radiol.

[7] Keall P.J., Mageras G.S., Balter J.M., Emery R.S., Forster K.M., Jiang S.B. (2006). The management of respiratory motion in radiation oncology report of AAPM Task Group 76. Med Phys.

[8] Speight R., Dubec M., Eccles C.L., George B., Henry A., Herbert T. (2021). IPEM topical report: guidance on the use of MRI for external beam radiotherapy treatment planning. Phys Med Biol.

[9] Alaya IB, Telmoudi M., Guesmi R., Mars M. (2021). Development of ACR quality control procedure for automatic assessment of spatial metrics in MRI. Biomed Res.

[10] Lewis B.C., Shin J., Maraghechi B., Quinn B., Cole M., Barberi E. (2022). Assessment of a novel commercial large field of view phantom for comprehensive MR imaging quality assurance of a 0.35T MRgRT system. J Appl Clin Med Phys.

[11] Stupic K.F., Ainslie M., Boss M.A., Charles C., Dienstfrey A.M., Evelhoch J.L. (2021). A standard system phantom for magnetic resonance imaging. Magn Reson Med.

[12] Ehman R.L., Felmlee J.P. (1989). Adaptive technique for high-definition MR imaging of moving structures. Radiology.

[13] Beck G., Stout J., Denolin V., Coenegrachts K., Herigault G. (2011). Real time velocity-based navigator triggering in the abdomen: initial results. Proc Intl Soc Magn Reson Med.

[14] Ichikawa S., Motosugi U., Wakayama T., Morisaka H., Funayama S., Tamada D. (2023). An intra-individual comparison between free-breathing dynamic MR imaging of the liver using stack-of-stars acquisition and the breath-holding method using cartesian sampling or view-sharing. Magn Reson Med Sci.

[15] Kajita K., Goshima S., Noda Y., Kawada H., Kawai N., Okuaki T. (2019). Thin-slice free-breathing pseudo-goldenangle radial stack-of-stars with gating and tracking T1-weighted acquisition: an efficient gadoxetic acidenhanced hepatobiliary-phase imaging alternative for patients with unstable breath holding. Magn Reson Med Sci.

[16] Van Lom K.J., Brown J.J., Perman W.H., Sandstrom J.C., Lee J.K. (1991). Liver imaging at 1.5 Tesla: Pulse sequence optimization based on improved measurement of tissue relaxation times. Magn Reson Imaging.

[17] Castellanos D.A., Jang J., Schidlow D.N., Brassaw K., Agudelo S., Heuvelink-Marck A. (2024). The impact of audiovisual breathing guidance on respiratory-triggered cardiac magnetic resonance cine imaging. Magn Reson Imaging.

[18] Grimbergen G., Hackett S.L., van Ommen F., van Lier A.L.H.M.W., Borman P.T.S., Meijers L.T.C. (2023). Gating and intrafraction drift correction on a 1.5 T MR-Linac: Clinical dosimetric benefits for upper abdominal tumors. Radiother Oncol.

