# Supplementary material for: A novel moving phantom insert for image quality assessment in magnetic resonance imaging
Source: Phys Imaging Radiat Oncol. 2025 Mar 7;34:100742. doi: 10.1016/j.phro.2025.100742 (PMC11957783; doi:10.1016/j.phro.2025.100742)
Supplement: Supplementary Data 1 [file mmc1.pdf]

Supplementary materials

Table S1. *Scan parameters of used sequences.*

	1	2	3	4	5
Sequence	T1-weighted Cartesian 3D Ultrafast Gradient Echo	T2-weighted PROPELLER Multi-Slice Turbo Spin Echo	T2-weighted Cartesian 3D Velocity-Based Triggered	T1-weighted Stack-of-stars 3D Vane XD	T2-weighted Cartesian 3D
Field of view (RL x AP x FH) [mm^3]	200 x 200 x 170	500 x 500 x 140	360 x 455 x 280	500 x 500 x 220	360 x 447 x 300
Flip angle	8	90	90	10	90
Slice orientation	Axial/ Coronal	Axial/ Coronal	Axial/ Coronal	Axial/ Coronal	Axial/ Coronal
Number of slices	34 – 85	40	233	147	250
Nominal slice thickness [mm]	2.0 – 5.0	2.0 – 5.0 (MR-sim) 3.5 (MR-linac)	2.4	3.0	2.4
Reconstructed voxel size [mm^3]	0.46 x 0.46 x 2.0 – 5.0	0.5 x 0.5 x 2.0 –5.0 (MR-sim) 3.5 (MR-linac)	0.78 x 0.78 x 1.2	0.78 x 0.78 x 1.5	0.78 x 0.78 x 1.2
TR [ms]	3.4	5000	2100	3.9	2100
TE [ms]	6.9	77-88 (MR-sim), 106 (MR-linac),	252	1.18	212
Total scan time [min:s]	00:33 – 01:19	02:00 – 05:00	02:21	06:01	03:20
Navigator triggering	No	Yes*/No	Yes	No	No
Motion compensation	No	Yes**	Yes***	Yes****	No

** Requires a software patch to define a 1D region of interest to allow for positional gating by setting an adjustable trigger-window width. Data acquisition is performed only when the 1D respiratory navigator (1D-RNAV) signal indicates that the phantom (within humans: the liver/lung interface) is within the end-expiration window. This sequence-combination compensates for in plane and through plane motion.*
*** Samples k-space using a set of radially directed blades. By oversampling the center of k-space the data for each new blade can be compared to the data from previous blades for consistency.*
**** Performs motion compensation by velocity-based 1D-RNAV triggering.*
***** Enables continuous acquisition of data and produces retrospective motion correction by exploiting data redundancy of central k-space by pseudo-golden-angle radial acquisition.*

Table S2. *Dimensions of wedges and line-pair structures.*

	Coronal wedge components	Axial wedge components	Coronal line-pair components
Amount	2	2	9
Length [mm]	60	60	40
Height [mm]	20	25	8
Width [mm]	12	12	1.5 – 2.0 – 2.5
Spaced apart [mm]	-	-	2.5 – 3.2 – 4.0
α_{wedge} [°]	18.4	22.6	-
$\alpha_{line-pair}$ [°]	-	-	45

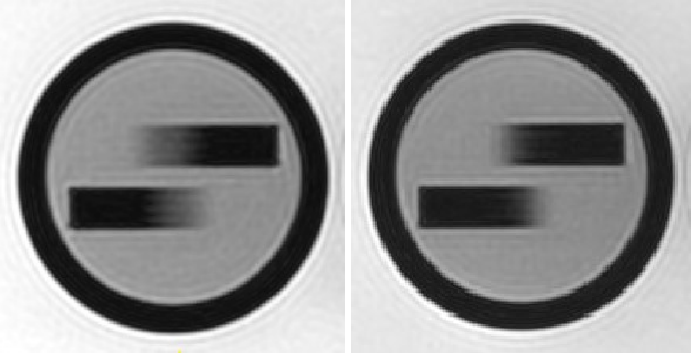

Figure S1. *Slice thickness image results for scans with a triggered T2w PROPELLER MS TSE sequence (TW = 6 mm) moving with a cosinusoidal[4] motion trajectory with 16 mm p2p with 0.25 Hz (left) and 0.22 Hz (right).*
